# Leukotriene inhibition in hamster periodontitis. A histochemical and morphometric study

**DOI:** 10.1155/S0962935192000504

**Published:** 1992

**Authors:** B. Baroukh, J. L. Saffar

**Affiliations:** Laboratoire de Biologie et Biomatériaux du Milieu Buccal et Osseux Groupe Physiopathologie Osseuse Faculté de Chirurgie Dentaire Université Paris-V, 1 rue Maurice Arnoux Montrouge 92120 France

## Abstract

The effects of leukotriene (LT) inhibition on gingival and adjacent bone compartments were assessed by using phenidone (100 mg/kg/d) and ketoconazole (50 mg/kg/d) given for 4 weeks to periodontitis-affected hamsters. In the gingiva the two agents significantly decreased PMNL recruitment and migration and increased the vascular lumen. At the bone level, they reduced significantly preosteoclast and osteoclast numbers but did not affect osteoclast activity. Phenidone had no action on periodontitis induced inhibition of bone formation; in contrast ketoconazole enhanced formation. As both phenidone and ketoconazole are unspecific LT inhibitors it cannot be ascertained that the effects observed were actually due to LT inhibition. However, phenidone and ketoconazole induced changes different from indomethacin used in previous studies to inhibit the cyclooxygenase pathway. These discrepancies suggest that LT inhibition occurred in the present study and that they participate in gingival inflammation and osteoclastic destruction during hamster periodontitis.


Mediators of Inflammation, 1, 335–339 (1992)

### Leukotriene inhibition in hamster periodontitis. A histochemical and morphometric study

B. Baroukh and J. L. Saffar[CA]

Laboratoire de Biologie et Biomatériaux du Milieu Buccal et Osseux, Groupe Physiopathologie Osseuse, Faculté de Chirurgie Dentaire, Université Paris-V, 1 rue Maurice Arnoux, 92120 Montrouge, France

[CA] Corresponding Author

**THE effects of leukotriene (LT) inhibition on gingival and adjacent bone compartments were assessed by using phenidone (100 mg/kg/d) and ketoconazole (50 mg/kg/d) given for 4 weeks to periodontitis-affected hamsters. In the gingiva the two agents significantly decreased PMNL recruitment and migration and increased the vascular lumen. At the bone level, they reduced significantly pre-osteoclast and osteoclast numbers but did not affect osteoclast activity. Phenidone had no action on periodontitis induced inhibition of bone formation; in contrast ketoconazole enhanced formation. As both phenidone and ketoconazole are unspecific LT inhibitors it cannot be ascertained that the effects observed were actually due to LT inhibition. However, phenidone and ketoconazole induced changes different from indomethacin used in previous studies to inhibit the cyclooxygenase pathway. These discrepancies suggest that LT inhibition occurred in the present study and that they participate in gingival inflammation and osteoclastic destruction during hamster periodontitis.**

**Key words:** Bone formation, Gingiva, Inflammation, Leukotriene, Osteoclast, Periodontitis, Resorption

### Introduction

5-lipoxygenase (5-LO) metabolites of arachidonic acid have been identified in high concentrations in inflamed gingival tissues.[1–3] However, their role in periodontal inflammation and their contribution to tissue destruction, if any, remains unknown.

It is only recently that systemic specific LT inhibitors[4] or receptor antagonists[5] have been developed. However, it was shown that some nonspecific agents also inhibited *in vivo* the 5-LO pathway. These include phenidone[6,7] and ketoconazole, an antimycotic drug.[8] In this paper it is reported that both phenidone and ketoconazole reduced gingival inflammation and osteoclastic resorption in a well-defined model of periodontitis.[9,10]

### Materials and Methods

Forty male golden hamsters (INRA, France), 6 weeks old, were used in this study. At the start of the experiment, ten animals were separated to constitute the control group; they were fed a standard diet for rodents (M25 Extralabo, Piétrement, France) for the whole experimental period. The other animals were fed the Keyes 2000 diet (U.A.R., France) which promotes bacterial plaque growth and subsequent periodontitis. After 8 weeks, the periodontitis-affected hamsters were separated into three equal groups: one group (group 2) remained treatment-free for the following 4 weeks; in group 3, phenidone (Sigma, USA), 50 mg/kg, diluted in distilled water was intraperitoneally injected twice a day [total dose 100 mg/kg/d[7]] for 4 weeks. In the last group (group 4), animals received 50 mg/kg/d o.s. of ketoconazole[8] diluted in a 5% aqueous gum arabic solution. This agent was kindly provided by Janssen Laboratories, France.

Animals were housed in a room exposed to natural light cycles (light, 10 h; dark, 14 h). Diet and water were provided *ad libitum*. At the end of the experimental period (12 weeks) all animals were killed under deep anaesthesia with a 8% chloral hydrate solution (Prolabo, France). Their left hemi-mandibles were rapidly dissected out and fixed by immersion for 24 h in cold (4°C) formaldehyde, 0.1 M calcium chloride in cacodylate buffer 0.2 M pH 7.4, rinsed in the same buffer and dehydrated. They were embedded without demineralization in glycol methacrylate. Four micron thick sections were cut in the horizontal plane and stained for acid phosphatase using naphthol AS-TR phosphate (Sigma, USA) and hexazotized pararosanilin (Sigma, USA). Non-osteoclastic acid phosphatase was inhibited with 50 mM L(+)-tartaric acid (Sigma, USA) added directly to the substrate solution. This procedure preserves tartrate resistant acid phosphatase, an enzyme specific of the osteoclastic lineage, and thus reveals only TRAP+ cells, namely preosteoclasts and osteoclasts. Sections were counterstained with toluidine blue (pH 3.8).

© 1992 Rapid Communications of Oxford Ltd

Morphometry was performed with the Morphomat 10 System (Zeiss, Germany) on three sections, 28 $\mu$m from each other, collected according to standards described elsewhere.[10] The following bone parameters were measured on the periosteal aspect of the lingual cortex between the first (m1) and second (m2) molars: (a) number of osteoclasts per mm of bone surface. We separately registered two morphologically different cell profiles: the active osteoclasts (in contact with the bone surface) and the inactive ones; (b) number of TRAP+ mononuclear precursors per mm of adjacent bone surface; (c) mean osteoclast-bone interface (OBI), an index of osteoclast activity, was obtained by dividing the resorption surface (in mm) by the number of active osteoclasts (in $\mu$m); (d) active resorption (in percentage of the bone surface measured); (e) reversal surface which materializes the metabolic intermediate phase between resorption and formation (in %); and (f) formation surface (in %).

On the same sections gingival inflammation was also assessed with the following parameters: (a) area of pocket epithelium surrounding m1 (in $mm^2$); (b) area of infiltrated connective tissue (ICT) identified as gingival subepithelial connective tissue containing inflammatory cells and exhibiting oedema and collagen rarefaction (in $mm^2$); (c) percentage of vessels in ICT; (d) mean vascular lumen (in $\mu m^2$); (e) number of PMNLs adherent to the vascular endothelium (in cells per vessel); (f) number of PMNLs in ICT (in cells for $mm^2$ of ICT); and (g) area of PMNL aggregated in the pocket space (in $mm^2$).

Data were statistically compared with one-way analyses of variance. When significant, Dunnett $t$-tests were performed. Results were accepted as significant at $p \leq 0.05$. They are given as mean $\pm$ SEM.

### Results

*Gingival inflammation parameters:* In the control animals, no periodontal pocket, a major component of periodontitis, was present. In contrast in all periodontitis-affected animals, a periodontal pocket always surrounded the roots of the first molar, the root surface was then covered by bacterial plaque. Aggregated PMNLs were interposed between the bacterial plaque and the pocket epithelium.

The increase in pocket epithelium area was marked between control and experimental animals ($p < 0.001$). The two agents did not change this parameter. Periodontitis dramatically increased ICT (12×, $p < 0.001$). Phenidone decreased it (−33% vs group 2), but not significantly. In contrast, ketoconazole further increased ICT (+30% vs group 2); this variation also was not significant. As a consequence the two treated groups were statistically different ($p < 0.01$) (Table 1).

Blood vessels occupied 5.92% of ICT in controls (Table 1). Periodontitis increased it up to 9.90%, although not significantly. The two treatments further increased the percentage of vessels in ICT, however only the phenidone group was significantly different from group 2 ($p < 0.05$). The mean vascular lumen increased significantly in untreated animals (+160% vs controls, $p < 0.01$). Both phenidone and ketoconazole induced an additional increase (Table 1), but only ketoconazole-treated hamsters differed from untreated ones ($p < 0.05$).

The number of PMNLs adherent to the vascular endothelium (Table 2) augmented in periodontitis-affected hamsters compared with controls but not significantly. The two treatments lowered this parameter (phenidone: −46% ($p < 0.02$) and ketoconazole: −37% ($p < 0.05$) vs group 2) below the control level. PMNLs in ICT were 8.7-fold higher in untreated than in control animals ($p < 0.001$). The two agents reduced them (phenidone: −59%, ketoconazole: −45% vs untreated animals, $p < 0.01$), but not to the control level. PMNL accumulation within the pocket space was also significantly reduced: −75% with phenidone ($p < 0.01$) and −54% with ketoconazole ($p < 0.05$) versus untreated animals (Table 2).

*Bone parameters:* Cell changes are outlined in Table 3. In control animals no osteoclast was found in the

**Table 1.** Infiltrated connective tissue (ICT) changes in the different groups. Vessels counted were those present in the ICT

| | Control | Periodontitis | Periodontitis + phenidone | Periodontitis + ketoconazole |
|---|---|---|---|---|
| ICT ($mm^2$) | 0.05 ± 0.02 | 0.63 ± 0.09$^c$ | 0.42 ± 0.09$^b$ | 0.83 ± 0.12$^{c,e}$ |
| Vascular area ($\mu m^2$) | 175.38 ± 20.19 | 462.50 ± 43.83$^b$ | 575.12 ± 51.47$^c$ | 616.89 ± 51.89$^{c,d}$ |
| Vessels in ICT (%) | 5.92 ± 1.20 | 9.90 ± 1.12 | 14.78 ± 1.37$^{b,d}$ | 11.56 ± 1.85$^a$ |

a, b and c different from controls at $p < 0.05$, $p < 0.01$, $p < 0.001$ respectively; d different from untreated periodontitis animals ($p < 0.01$) and e from phenidone-treated hamsters ($p < 0.01$).

**Table 2.** Variations in PMNLs in the gingival compartment of the diseased periodontium. The main effect of both phenidone and ketoconazole concerned PMNL recruitment in the inflamed site

| | Control | Periodontitis | Periodontitis + phenidone | Periodontitis + ketoconazole |
|---|---|---|---|---|
| Marginating PMNLs (cells/vessel) | $0.86 \pm 0.15$ | $1.28 \pm 0.18$ | $0.69 \pm 0.08^{d}$ | $0.80 \pm 0.17^{c}$ |
| PMNLs in ICT (cells/mm$^2$) | $60.56 \pm 26.34$ | $531.75 \pm 92.86^{b}$ | $215.75 \pm 27.57^{e}$ | $293.96 \pm 37.20^{a,e}$ |
| PMNLs around plaque (mm$^2$) | $0.00 \pm 0.00$ | $0.11 \pm 0.03^{b}$ | $0.03 \pm 0.01^{e}$ | $0.05 \pm 0.02^{c}$ |

a and b different from group controls at $p < 0.05$, $p < 0.001$; c, d and e different from affected untreated animals at $p < 0.05$, $p < 0.02$ and $p < 0.01$, respectively.

**Table 3.** Changes in osteoclastic cells along the periosteal bone surface adjacent to ICT. Both preosteoclasts and osteoclasts were reduced, although their decreases did not reach the control levels

| | Control | Periodontitis | Periodontitis + phenidone | Periodontitis + ketoconazole |
|---|---|---|---|---|
| Total osteoclasts (cells/mm) | $0.00 \pm 0.00$ | $7.87 \pm 1.05^{b}$ | $4.63 \pm 0.73^{a,c}$ | $4.55 \pm 0.68^{a,c}$ |
| Active osteoclasts (cells/mm) | $0.00 \pm 0.00$ | $5.30 \pm 0.49^{b}$ | $3.26 \pm 0.23^{a,c}$ | $3.40 \pm 0.79^{a,c}$ |
| Preosteoclasts (cells/mm) | $0.61 \pm 0.61$ | $35.36 \pm 2.55^{b}$ | $10.90 \pm 2.05^{a,d}$ | $15.14 \pm 1.66^{a,d}$ |

a and b different from group controls at $p < 0.01$ and $p < 0.001$; c and d different from affected untreated animals at $p < 0.02$ and $p < 0.001$.

reference bone segment. Periodontitis caused a dramatic increase in the total number of osteoclasts. They were similarly reduced by the two agents ($p < 0.02$), but remained at a rather high level compared with controls. Active profiles decreased in the same proportion with the two agents ($p < 0.02$). Inactive profiles also decreased but their variations were not significant. The ratio active/inactive profiles was 2.06 in untreated animals, 2.37 with phenidone and 2.9 with ketoconazole. The mean OBI was not statistically modified by the treatments. Preosteoclasts increased 58 times in untreated animals compared with controls ($p < 0.001$).

Phenidone and ketoconazole reduced preosteoclasts by 69% and 57% respectively ($p < 0.001$).

Surface activity changes are outlined in Table 4. In the untreated animals, resorption occupied 15.17% of the bone surface. The two agents reduced it by 50% ($p < 0.01$). Reversal increased in untreated animals by 9.4-fold ($p < 0.001$ vs controls). It was not significantly modified with phenidone, in contrast ketoconazole decreased it (−34%, $p < 0.01$). The discrepancy between the treated groups made them significantly different ($p < 0.02$).

Formation was the main bone surface activity in controls; periodontitis almost completely abolished it (−88%, $p < 0.01$). Unlike phenidone, ketoconazole partially reversed the effect of periodontitis as a conspicuous increase in formation occurred in this treatment group ($p < 0.05$ vs group 2). As a consequence, the action of the two agents was significantly different ($p < 0.05$).

Some gingival and bone parameters were statistically related. The more significant relationships concerned the number of PMNLs in ICT and

**Table 4.** Variations in bone surface activity parameters. The two agents had opposite actions on formation: phenidone had no effect on the uncoupling due to periodontitis while ketoconazole induced a significant increase in formation

| | Control | Periodontitis | Periodontitis + phenidone | Periodontitis + ketoconazole |
|---|---|---|---|---|
| Resorption | $0.00 \pm 0.00$ | $15.17 \pm 1.81^{a}$ | $7.78 \pm 1.07^{a,d}$ | $7.18 \pm 1.65^{a,d}$ |
| Reversal | $4.81 \pm 2.85$ | $45.21 \pm 4.06^{b}$ | $50.27 \pm 6.98^{b}$ | $29.68 \pm 6.92^{a,f}$ |
| Formation | $35.80 \pm 6.25$ | $4.08 \pm 2.67^{a}$ | $5.50 \pm 3.55^{a}$ | $25.82 \pm 9.01^{c,e}$ |

a and b different from group controls at $p < 0.01$ and $p < 0.001$; c and d different from affected untreated animals at $p < 0.05$ and $p < 0.01$; e and f different from phenidone-treated hamsters at $p < 0.05$ and $p < 0.02$.

respectively the extent of resorption ($r = 0.66$; $p = 0.0001$), and the total number of osteoclasts ($r = 0.49$; $p = 0.008$). Preosteoclasts and total osteoclasts were also statistically related ($r = 0.65$; $p = 0.0001$).

### Discussion

Taken together the results show that the two unspecific LT inhibitors had conspicuous actions on both gingival and bone changes due to periodontitis. This suggests that LTs might be involved in hamster periodontitis, a statement in agreement with observations showing that LT concentrations increased in gingival tissues during periodontitis.[1–3] At the gingival level the more striking action of the two agents consisted in a prominent decrease in PMNLs in the three compartments being investigated, viz adherent to blood vessel endothelium, infiltrating the gingival connective tissue, and aggregated in the pocket space. This was primarily due to a marked depression in PMNL recruitment and penetration in the site as shown by the reduction in adherent PMNLs. Therefore, phenidone and ketoconazole inhibited PMNL chemotaxis, an action generally assigned to $LTB_4$.[11] $LTB_4$ would be released either by endothelial cells[12] or by cells of the extravascular compartment such as PMNLs,[13] monocytes and macrophages,[14,15] and epithelial cells.[16] Besides $LTB_4$, stimulated endothelial cells would release other unidentified lipoxygenase-derived PMNL chemoattractants.[17] The reduction in PMNLs aggregated in the pocket space was in proportion more marked than in ICT although the mass of bacteria was not modified (data not shown). As epithelial cells release $LTB_4$ when properly activated,[16] phenidone and ketoconazole may have disturbed an epithelial chemotactic gradient governing PMNL emigration towards bacteria.

The partial inhibition of PMNL chemotaxis indicates that several mediators attracted PMNLs in the gingival tissues. Besides LTs, inflammatory and resident cells release other potent PMNL chemoattractants such as IL-1$\beta$ and TNF,[18,19] C5a,[20] C3b,[19] serotonin.[17] Prostanoids also promote PMNL recruitment,[21] in this model cyclooxygenase (CO) inhibition reduced PMNL aggregation in the pocket.[22]

Oedema is another component of gingival inflammation possibly mediated by LTs. Inhibition of the LT-dependent endothelial leakage may have led to the reduction in ICT in the phenidone-treated animals. However, the increase in ICT in the ketoconazole-treated group is puzzling in this view. Phenidone and ketoconazole induced vasodilatation, in fact $LTC_4$ and $LTD_4$ are vasoconstrictors.[11] The higher effectiveness of ketoconazole might be due to its wider spectrum of activity as it also inhibits $TXA_2$,[8,23] a vasoconstrictor.[24] The increase in the percentage of vessels in the two treated groups might be due to vessel proliferation in the treated groups, however as $LTC_4$ is a promoter of endothelial cell proliferation,[25] such an effect can be excluded under LT inhibition. Alternatively, the increase in vessels might only be relative with phenidone; larger vessels in less ICT may induce an apparent increase in vessels. With ketoconazole the potential incidence of the more intense vasodilatation on this parameter was possibly lessened by the increase in ICT.

At the bone level the more conspicuous effect was the reduction in osteoclastic resorption. This result is consistent with the observation that $LTB_4$ and the peptido-LTs stimulated bone resorption *in vitro*,[26] through modifications in the concentration of osteoblast intracellular calcium.[27] These observations support the contention that LTs directly stimulate resorption. However, the effects on resorption might be indirect. The decrease in differentiated osteoclasts was indeed related to the reduction in PMNLs present at the site taken as an index of gingival inflammation, suggesting that the higher the inflammatory reaction, the greater the number of osteoclasts in the site and the higher the level of resorption. On the other hand, the two agents may have inhibited in cascade the release of other mediators of resorption. As a matter of fact, $LTB_4$ and $LTD_4$ are involved in the production of IL-1 by endotoxin-stimulated monocytes[28] via a mobilization of intracellular calcium.[29] The role of IL-1 as a promotor of resorption is well documented. However, the role, if any, of LTs in resorption is still unknown. The relationship found between osteoclasts and preosteoclasts, which is a characteristic of hamster periodontitis,[10] indicates that phenidone and ketoconazole did not impair the differentiation of preosteoclasts into osteoclasts, and the process of multinucleation.

The main problem with regard to the present results is whether the two agents inhibited only LTs or both 5-LO and CO pathways. Indeed, Blackwell and Flower[6] stated that phenidone inhibited the two pathways in the same extent. However, Carlson *et al.*[7] showed in the rat that phenidone had a potent inhibitory activity against 5-LO but was only a weak CO inhibitor. In this model of periodontitis, when the bone effects of the disease were inhibited with the CO inhibitor indomethacin, formation was restored beyond the control level[22,30] showing that prostanoids were involved in the uncoupling between resorption and formation, another characteristic feature of periodontitis.[9] Coupling refers of the events ensuring the focal recruitment and proliferation of osteoblast precursors and the expression of the osteoblast phenotype.[31] In the present study phenidone was quite unable to activate formation indicating that it had no action on prostanoid-induced uncoupling. This data suggests that LTs are actually not involved in periodontitis coupling disturbances. On the other hand in two non-inflammatory models of bone resorption, indomethacin lowered the ratio active/inactive osteoclasts.[32,33] Conversely, in the present study the ratio was increased, slightly with phenidone and more markedly with ketoconazole. Indomethacin also reduced osteoclast activity (Leroux and Saffar, submitted for publication) while phenidone and ketoconazole did not. Therefore the effects on osteoclast parameters of indomethacin on one hand and phenidone and ketoconazole on the other hand were quite different. In addition, in a non-inflammatory model of resorption, we observed with a specific 5-LO inhibitor, the same changes in TRAP+ preosteoclasts and osteoclasts as in the present study (C. Franchi, unpublished data). These observations led us to assume that, at least at the bone level, the changes induced by phenidone were not due to prostanoid inhibition but rather were LT related. Ketoconazole is a less effective inhibitor of LT synthesis than phenidone,[8] it also inhibits $TXA_2$ synthesis probably through an interaction with the cytochrome P-450[8,23] It may thus be anticipated that ketoconazole inhibited concomitantly several mediators, which might explain some discrepancies between phenidone and ketoconazole at both gingival and bone levels. In particular, like indomethacin, ketoconazole induced an increase in formation, although to a lesser extent. To the authors' knowledge the precise action of $TXA_2$ on bone formation has not been determined, thus an effect of ketoconazole on formation through $TXA_2$ inhibition remains speculative. On the other hand, ketoconazole interferes with the metabolism of 1,25 $(OH)_2D_3$ in osteoblast-like cells[34] and this action might contribute to the increase in formation in this group.

### References

1. Scott S, Odle B, Offenbacher S. Inflamed periodontal tissues contain high levels of leukotriene B4. *J Dent Res* 1985: **64**: 377 (abstract).
2. El Attar TMA, Lin HS, Killoy WJ, Vanderhoek Y, Goodson JM. Hydroxy fatty acids and prostaglandin formation in diseased human periodontal pocket tissue. *J Period Res* 1986; **21**: 169–176.
3. Offenbacher S, Odle BM, Van Dyke TE. Steady state levels of leukotrienes B4, C4, D4, and E4 in periodontal tissues. *J Dent Res* 1986; **65**: 756 (abstract).
4. Tateson JE, Randall RW, Reynolds CH, *et al.* Selective inhibition of arachidonate 5-lipoxygenase by novel acetohydroxamic acids: biochemical assessment *in vitro* and *ex vivo*. *Br J Pharmacol* 1988; **94**: 528–539.
5. Snyder DW, Fleisch JH. Leukotriene receptor antagonists as potential therapeutic agents. *Ann Rev Pharmacol Toxicol* 1989; **29**: 123–143.
6. Blackwell GJ, Flower RJ. 1-Phenyl-3-pyrazolidone: an inhibitor of cyclo-oxygenase and lipoxygenase pathways in lung and platelets. *Prostaglandins* 1978; **16**: 417–425.
7. Carlson RP, O'Neill-Davis L, Chang J, Lewis AJ. Modulation of mouse ear edema by cyclooxygenase and lipoxygenase inhibitors and other pharmacologic agents. *Agents Actions* 1985; **17**: 197–204.
8. Beetens JR, Loots W, Somers Y, Coene MC, de Clerck F. Ketoconazole inhibits the biosynthesis of leukotrienes *in vitro* and *in vivo*. *Biochem Pharm* 1986; **35**: 883–891.
9. Makris GP, Saffar JL. Disturbances in bone remodeling during the progress of hamster periodontitis. A morphological and quantitative study. *J Period Res* 1985; **20**: 411–420.
10. Baroukh B, Saffar JL. Identification of osteoclasts and their mononuclear precursors. A comparative histological and histochemical study in hamster periodontitis. *J Period Res* 1991; **26**: 161–166.
11. Dahlen SE, Björk J, Hedqvist P, *et al.* Leukotrienes promote plasma leakage and leukocyte adhesion in postcapillary venules: *in vivo* effects with relevance to the acute inflammatory response. *Proc Natl Acad Sci USA* 1981; **78**: 3887–3891.
12. Nolan KD, Keagy BA, Ramadan FM, Johnson G, Henke DC. Endothelial cells can synthesize leukotriene-B4. *J Vasc Surg* 1990; **12**: 298–304.
13. Ford-Hutchinson AW, Bray MA, Doig MV, Shipley ME, Smith MJH. Leukotriene B, a potent chemokinetic and aggregating substance released from polymorphonuclear leukocytes. *Nature* 1980; **286**: 264–265.
14. Fels AOS, Pawlowski NA, Cramer EB, King ZA, Scott WA. Human alveolar macrophages produce leukotriene B4. *Proc Natl Acad Sci USA* 1982; **79**: 7866–7870.
15. Williams JD, Czop JK, Austen KF. Release of leukotrienes by human monocytes on stimulation of their phagocytic receptor for particulate activators. *J Immunol* 1984; **132**: 3034–3040.
16. Brain SD, Camp RDR, Leigh IM, Ford-Hutchinson AW. The synthesis of leukotriene B4-like material by cultured human keratinocytes. *J Invest Dermatol* 1982; **78**: 328 (abstract).
17. Charles A, Rounds S, Farber HW. Neutrophil chemoattractant production by cultured serotonin-stimulated bovine and human endothelial cells. *Am J Physiol* 1991; **261**: L133–L139.
18. Moser R, Schleiffenbaum B, Groscurth P, Fehr J. Interleukin 1 and tumor necrosis factor stimulate human vascular endothelial cells to promote transendothelial neutrophil passage. *J Clin Invest* 1989; **83**: 444–455.
19. Ward PA, Marks RM. The acute inflammatory reaction. *Curr Opin Immunol* 1989; **2**: 5–9.
20. McMillan RM, Foster SJ. Leukotriene B4 and inflammatory disease. *Agents Actions* 1988; **24**: 114–119.
21. Palder SB, Huval W, Lelcuk S, *et al.* Reduction of polymorphonuclear leukocyte accumulation by inhibition of cyclooxygenase and thromboxane synthase in the rabbit. *Surgery* 1986; **99**: 72–80.
22. Carter-Bartlett P, Dersot JM, Saffar JL. Periodontal and femoral bone status in periodontitis-affected hamsters receiving a high dose indomethacin treatment. *J Biol Buccale* 1989; **17**: 93–101.
23. Dunham BM, Hechtman HB, Valeri CR, Shepro D. Antiinflammatory agents inhibit microvascular permeability induced by leukotrienes and by stimulated human neutrophils. *Microcirc Endoth Lymph* 1984; **1**: 465–489.
24. Davies P, Bailey PJ, Goldenberg MM. The role of arachidonic acid oxygenation products in pain and inflammation. *Ann Rev Immunol* 1984; **2**: 335–357.
25. Modat G, Muller A, Mary A, Bonne C. LTC4, but not LTB4, binds vascular endothelial cells and promotes their proliferation *in vitro*. *Ann New York Acad Sci* 1988; **524**: 414–416.
26. Meghji S, Sandy JR, Scutt AM, Harvey W, Harris M. Stimulation of bone resorption by lipoxygenase metabolites of arachidonic acid. *Prostaglandins* 1988; **36**: 139–149.
27. Sandy JR, Meikle MC, Martin BR, Farndale RW. Leukotriene B4 increases intracellular calcium concentration and phosphoinositide metabolism in mouse osteoblasts via cyclic adenosine 3',5'-monophosphate-independent pathways. *Endocrinology* 1991; **129**: 582–590.
28. Rola-Pleszczynski M, Lemaire I. Leukotrienes augment interleukin 1 production by human monocytes. *J Immunol* 1985; **6**: 3958–3961.
29. Tatsuno I, Saito H, Chang KJ, Tamura Y, Yoshida S. Comparison of the effect between leukotriene B4 and leukotriene B5 on the induction of interleukin 1-like activity and calcium mobilizing activity in human blood monocytes. *Agents Actions* 1990; **29**: 324–327.
30. Saffar JL, Lasfargues JJ. A histomorphometric study of the effect of indomethacin and calcitonin on bone remodelling in hamster periodontitis. *Archs Oral Biol* 1984; **29**: 555–558.
31. Parfitt AM. The cellular basis of bone remodeling: the quantum concept reexamined in light of recent advances in the cell biology of bone. *Calcif Tissue Int* 1984; **36**: 37–45.
32. Saffar JL, Leroux P. Role of prostaglandins in bone resorption in a synchronized remodeling sequence in the rat. *Bone* 1988; **9**: 141–145.
33. Lasfargues JJ, Saffar JL. Effects of prostaglandin inhibition on the bone activities associated with the spontaneous drift of molar teeth in the rat. *Anatomical Record* 1992; (in press).
34. Reinhardt TA, Horst RL. Ketoconazole inhibits self-induced metabolism of 1, 25-dihydroxyvitamin D3 and amplifies 1,25-dihydroxyvitamin D3 receptor up-regulation in rat osteosarcoma cells. *Arch Biochem Biophys* 1989; **272**: 459–465.

**Received 30 June 1992; accepted in revised form 30 July 1992**
